# Return on Investment in Electronic Health Records in Primary Care Practices: A Mixed-Methods Study

**DOI:** 10.2196/medinform.3631

**Published:** 2014-09-29

**Authors:** Yeona Jang, Michel A Lortie, Steven Sanche

**Affiliations:** ^1^McGill UniversityDesautels Faculty of ManagementMontreal, QCCanada; ^2^St Mary's Research CentreMontreal, QCCanada

**Keywords:** return on investment in electronic health records, cost recovery from EHR implementation, ROI indicator, physician satisfaction with EHR, primary care practices

## Abstract

**Background:**

The use of electronic health records (EHR) in clinical settings is considered pivotal to a patient-centered health care delivery system. However, uncertainty in cost recovery from EHR investments remains a significant concern in primary care practices.

**Objective:**

Guided by the question of “When implemented in primary care practices, what will be the return on investment (ROI) from an EHR implementation?”, the objectives of this study are two-fold: (1) to assess ROI from EHR in primary care practices and (2) to identify principal factors affecting the realization of positive ROI from EHR. We used a break-even point, that is, the time required to achieve cost recovery from an EHR investment, as an ROI indicator of an EHR investment.

**Methods:**

Given the complexity exhibited by most EHR implementation projects, this study adopted a retrospective mixed-method research approach, particularly a multiphase study design approach. For this study, data were collected from community-based primary care clinics using EHR systems.

**Results:**

We collected data from 17 primary care clinics using EHR systems. Our data show that the sampled primary care clinics recovered their EHR investments within an average period of 10 months (95% CI 6.2-17.4 months), seeing more patients with an average increase of 27% in the active-patients-to-clinician-FTE (full time equivalent) ratio and an average increase of 10% in the active-patients-to-clinical-support-staff-FTE ratio after an EHR implementation. Our analysis suggests, with a 95% confidence level, that the increase in the number of active patients (*P*=.006), the increase in the active-patients-to-clinician-FTE ratio (*P*<.001), and the increase in the clinic net revenue (*P*<.001) are positively associated with the EHR implementation, likely contributing substantially to an average break-even point of 10 months.

**Conclusions:**

We found that primary care clinics can realize a positive ROI with EHR. Our analysis of the variances in the time required to achieve cost recovery from EHR investments suggests that a positive ROI does not appear automatically upon implementing an EHR and that a clinic’s ability to leverage EHR for process changes seems to play a role. Policies that provide support to help primary care practices successfully make EHR-enabled changes, such as support of clinic workflow optimization with an EHR system, could facilitate the realization of positive ROI from EHR in primary care practices.

## Introduction

### Context

The use of electronic health records (EHR) in clinical settings is widely recommended as an innovation enabler with potential benefits of reducing health care costs, while improving quality and safety, and is considered central to achieving patient-centered health care [1-4]. As a wide array of EHR projects have been implemented within various health care settings, the health care field is rich with volumes of work examining the benefits of EHR. However, the existing literature reports mixed results in benefits realized from EHR implementation [5,6]. Such mixed results suggest that the implementation of EHR systems does not automatically guarantee the conversion of potential benefits into realized benefits.

The implementation of EHR systems within primary care practices is seen as particularly complex [7-10], with physicians and other staff in primary care practices citing obstacles such as difficulty in adapting to the significant changes in workflow and the time commitment required to learn to use the new software while prioritizing patient care [11-14]. While there is a growing body of evidence that EHR can be a valuable tool for improving quality of care and patient safety with relatively positive perceptions about EHR benefits [15-17], uncertainty about cost recovery of an EHR investment remains a significant concern in primary care practices [7,8,18,19]. Various studies on EHR impact and adoption also raise the need for cost-benefit analysis of EHR investments [5,20]. Thus, this study seeks to assess the return on investment (ROI) from an EHR implementation in primary care settings, aiming to complement the current insights on cost recovery concerns in existing literature.

### Measurement

Return on investment is a common approach to measuring rates of return on money invested, in terms of increased profit attributable to the investment. A standard ROI is defined as follows:

ROI = (Gain from investment - Cost of investment)/Cost of investment

Results reported by various studies regarding ROI from EHR systems in primary care settings are mixed [21-25]. Most of the existing literature used a bottom-up approach identifying specific cost-saving areas and collecting the data on financial savings made in these areas attributable to EHR systems. However, EHR is a process-enabling information technology (IT) that offers the opportunity to streamline information-intensive workflow, remove manual hand-off of data and information, and facilitate coordination—thus facilitating the execution of entire business processes rather than individual tasks. Due mainly to the context-sensitive nature of benefits realization from a process-enabling IT such as EHR and the scarcity of detailed financial data relating to gains and/or savings directly attributable to an EHR system in primary care clinics, this study used break-even-point analysis as an indicator of ROI, instead of standard ROI analysis.

The break-even point of an EHR investment is defined as the number of months it takes a clinic to recover the cost of the EHR system and other associated implementation costs, with increased revenues and/or decreased expenses. Increases in revenues and/or decreases in expenses are assessed by considering net revenues during three distinct periods of time: pre-EHR, peri-EHR, and post-EHR. The pre-EHR period is defined as the full fiscal year before the implementation of an EHR system started. The peri-EHR period is defined as the fiscal year(s) containing the EHR implementation period (ie, during EHR implementation). If the peri-EHR period covers more than one fiscal year, the net revenue is averaged over these fiscal years. The post-EHR period is defined as the full fiscal year following the end of the peri-EHR period.

To calculate the break-even point of implementing an EHR system in a clinic, the cost of EHR implementation is set equal to the difference in the clinic’s net revenue between the pre-EHR and peri-EHR periods, plus the difference in the clinic’s net revenue between the pre-EHR and post-EHR periods, as summarized in the following formula:

C _*EHR*_=[(NR _*peri*_−NR _*pre*_)/12]*M _*imp*_ + [(NR _*post*_−NR _*pre*_)/12] * (M _*break-even*_−M _*imp*_)

In this formula: *C*_*EHR*_=cost of EHR implementation, *NR*_*peri*_=annual clinic net revenue in the peri-EHR period*, NR*_*pre*_=annual clinic net revenue in the pre-EHR period, *NR*_*post*_=annual clinic net revenue in the post-EHR period, *M*_*imp*_=the number of months taken to complete the EHR implementation in the clinic, and *M*_*break-even*_=months to break even. The net revenue of a clinic is defined as the sum of the physicians’ billings for work done in the clinic minus any expenses that the clinic pays to maintain its ongoing practice. In the case where the months to break even were less than the months of EHR implementation, in other words, the net revenue difference between pre-EHR and peri-EHR periods is large enough to recover the cost of EHR implementation, the formula was adjusted by setting the cost of EHR implementation equal to the difference in a clinic’s net revenue between the pre-EHR and peri-EHR periods, or:

*C*_*EHR*_ =[(*NR*_*peri*_ – *NR*_*pre*_)/12]* *M*_*break-even*_

### Objectives

Guided by the research question “When implemented in primary care practices, what will be the ROI from an EHR implementation?”, the objectives of this research are twofold: (1) to assess the ROI from an EHR implementation in primary care practices by measuring the time required to recover the cost of converting a clinic from a paper-based environment to an EHR-enabled environment and (2) to identify principal factors affecting the realization of a positive ROI from an EHR implementation in primary care practices. Such ROI information related to cost recovery of an EHR investment would be helpful to both clinics considering implementing EHR systems and to policy makers designing EHR-adoption funding programs and policies.

## Methods

### Sample

Community-based, primary care clinics meeting the following four eligibility criteria were recruited for this study on ROI from EHR in primary-care settings. First, this study focused on community-based, primary care clinics. Thus, specialty care clinics and walk-in clinics were excluded. Second, clinics were required to have implemented EHR systems. Third, clinics were required to have been paper-based in the past, in order to ensure that the comparison between pre-EHR and post-EHR implementation performance was possible for the ROI calculation. Fourth, clinics were required to provide operational and financial data necessary to calculate ROI, as well as the information on challenges and opportunities that they had experienced both during and after the EHR implementation.

The research team contacted 132 randomly selected community-based, primary care clinics in Canada that met the first two eligibility criteria for recruitment to the study. Of the 132 clinics, 62 clinics declined to participate, mostly citing time constraints. Of the 70 clinics remaining, 34 clinics were not eligible, mainly because they were unable to provide the operational and financial data necessary to calculate ROI. Of the 36 eligible clinics, 19 clinics later declined to participate, due mainly to time constraints. Thus, data were collected from a total of the 17 eligible clinics, resulting in the study participation rate of 13%, which is relatively consistent with typical participation rates of family physicians reported in other studies involving interviews and observations [26]. No statistically significant differences were observed between participating and non-participating primary care clinics in terms of geographic location (*P*=.315), the number of physicians or other clinicians (*P*<.001), or the number of patients per physician (*P*=.192). Table 1 summarizes the basic statistics on the size of the sampled clinics. We used Full Time Equivalent (FTE) in comparing the size of primary care clinics.

**Table 1:** Basic statistics on the size of a primary care clinic in the study.

		Average	SD	Median	Minimum	Maximum
**Clinic size: clinician FTEs**						
	Pre-EHR period	3.4	2.6	3.0	1.0	8.5
	Post-EHR period	3.6	2.4	3.0	0.8	8.0
**Clinic size: clinical support staff FTEs**						
	Pre-EHR period	3.4	2.9	2.8	1.0	12.0
	Post-EHR period	4.2	3.1	3.0	0.9	12.0

### Methodology

Given the complexity exhibited by most EHR implementation projects, this study used a mixed-method research approach, particularly a multiphase study design [27]. By combining quantitative and qualitative data, mixed-method research can provide a fuller understanding of the complex and multidimensional world of primary care clinics than would otherwise be achieved using either approach alone.

In the quantitative study phase, questionnaire modules were designed, based on prior research in the existing literature [28-33], to collect data on EHR implementation costs, EHR functionalities in use, physician satisfaction with EHR, and physicians’ perceptions about the impact of EHR on operational efficiency and on quality of care. Each clinic respondent was required to complete the study instruments using the online questionnaires or researcher-assisted telephone questionnaires. The minimum financial data required for the study include clinic revenue and clinic net revenue as well as EHR implementation cost that consisted of EHR software costs, hardware costs, support costs, and labor costs associated with EHR system implementation and training, in the three different periods—before EHR implementation, during EHR implementation, and after EHR implementation. The minimum operational data required for this ROI study include the number of active patients, clinician FTEs, and clinical support staff FTEs, in the same three periods. The lead researcher served as dedicated support liaison for clinics, in order to ensure that the costs of the EHR implementation, as well as other financial and operational data before and after EHR implementation, were abstracted from clinic records in a consistent fashion. In the subsequent qualitative study, semistructured interviews and observations were conducted with clinic staff and physicians identified as responsible for such functions as patient appointment management, patient record management, test results management, patient encounters, and billing, to assess factors affecting the realization of a positive ROI from an EHR implementation in primary care practices.

The data collected from 17 sampled primary care clinics were documented and analyzed using statistical analysis and grounded theory [34]. As break-even points were analyzed, we compared those clinics that were successful in realizing a positive ROI from EHR implementation to those that were less successful, in an attempt to identify principal factors impacting the realization of positive ROI from EHR. In particular, we used linear regression analysis to estimate the relationships of the outcome variable “break-even point” with the explanatory variables that include the codes identified from the qualitative data through the coding process.

## Results

### Analysis of Break-Even Point as Indicator of Return on Investment

#### Overview

Our analysis suggests that the sampled primary care clinics typically recovered their investment in EHR within an average of 10 months (95% confidence interval: 6.2 months, 17.4 months), seeing more patients with improved active-patients-to-clinician-FTE and active-patients-to-clinical-support-staff-FTE ratios in the post-EHR implementation period.

#### Change in Clinic Net Revenue After Implementation of Electronic Health Records

Once an EHR system is implemented, a key factor that impacts the time required to achieve cost recovery from the EHR investments is clinic net revenue. With respect to how clinics fared financially upon adopting EHR systems, all but one of the primary care clinics in our study achieved an increase in clinic net revenue in the post-EHR period, as shown in Table 2.

**Table 2:** Percent changes in clinic revenues, net revenues, and clinician FTEs between the pre-EHR and post-EHR periods.

Clinic #	Percent change between the pre-EHR and post-EHR periods (in ascending order by percent change in number of clinician FTEs), %		
	In number of clinician FTEs	In clinic revenue	In clinic’s net revenue
Clinic 1	-29	23	23
Clinic 2	-20	-28	22
Clinic 3	-14	27	4
Clinic 4	-2	29	26
Clinic 7	0	55	9
Clinic 5	0	50	63
Clinic 9	0	33	8
Clinic 10	0	31	28
Clinic 8	0	23	28
Clinic 11	0	19	16
Clinic 6	0	3	15
Clinic 12	0	-10	20
Clinic 13	0	-15	-30
Clinic 14	10	120	116
Clinic 15	47	223	227
Clinic 16	53	103	98
Clinic 17	329	603	845
Average	22	76	89

**Figure 1:**
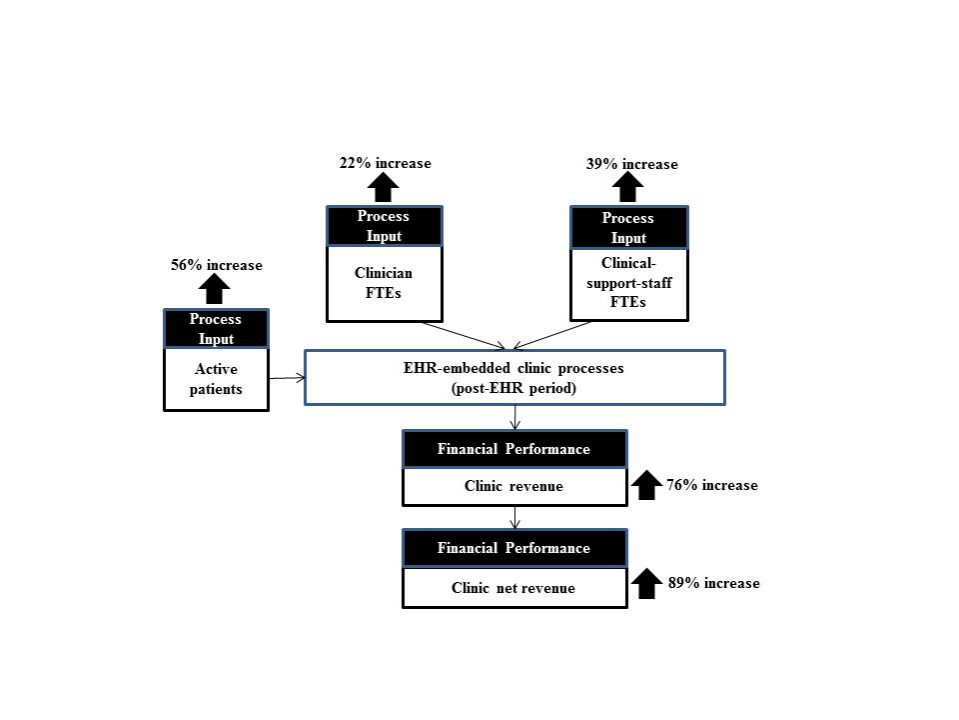
Average percent changes in active patient count, clinician FTE count, clinical support staff FTE count, clinic revenue, and clinic net revenue between the pre-EHR and post-EHR periods.

In addition to clinic net revenue, the sampled clinics showed, on average, positive increases in active patient count, clinician count, clinical support staff count, and clinic revenue in the post-EHR implementation period. These increases are summarized in Figure 1.

**Figure 2:**
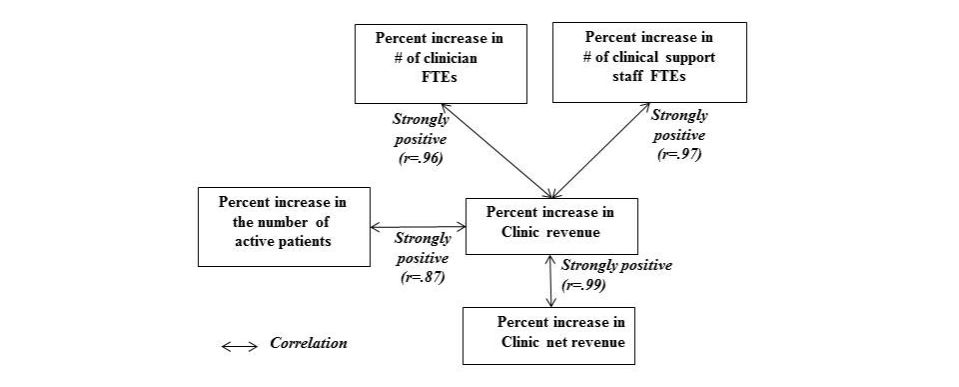
Correlations (r-values): clinic net revenue, clinic revenue, active patient count, clinician FTE count, and clinical support staff FTE count.

Percent increase in clinic net revenue between the pre-EHR and post-EHR periods showed a very strong positive correlation with percent increase in clinic revenue in the same periods (*r*=.99). Percent increase in clinic revenue showed a strong positive correlation with percent increase in the number of active patients (*r*=.87). It also showed a strong positive correlation with percent increase in the number of clinician FTEs, as well as with the number of clinical-support-staff FTEs (*r*=.96 and *r*=.97, respectively). These correlation coefficients (*r* values) are summarized in Figure 2.

#### Percent Changes of Counts After Implementation—Not Linearly Proportional to One Another

**Figure 3:**
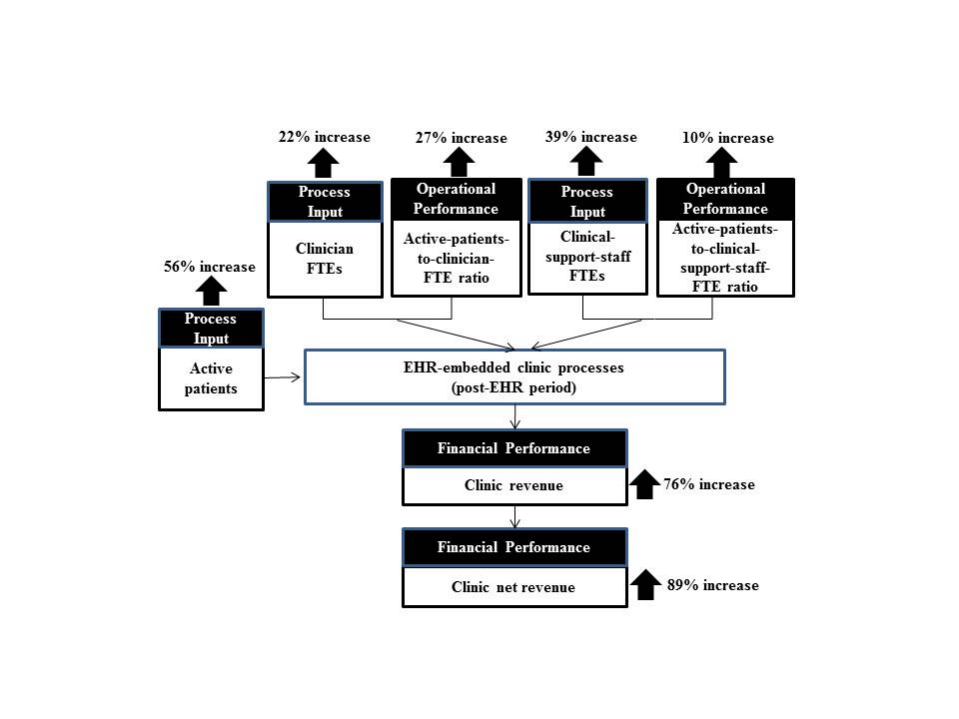
Average percent changes in a clinic’s operational efficiency and financial performance between the pre-EHR and post-EHR periods.

Interestingly, the percent increases in active patient count, clinician FTE count, and clinical support staff FTE count are not linearly proportional to one another. An average active-patient-count increase of 56% was handled by an average 22% increase for clinician FTEs and an average 39% increase for clinical-support-staff FTEs. This finding suggests change in operational efficiency after EHR implementation, with respect to the active-patients-to-clinician-FTE ratio and the active-patients-to-clinical-support-staff-FTE ratio. The sampled clinics showed an average increase of 27% in the active-patients-to-clinician-FTE ratio and an average increase of 10% in the active-patients-to-clinical-support-staff-FTE ratio, as illustrated in Figure 3.

**Figure 4:**
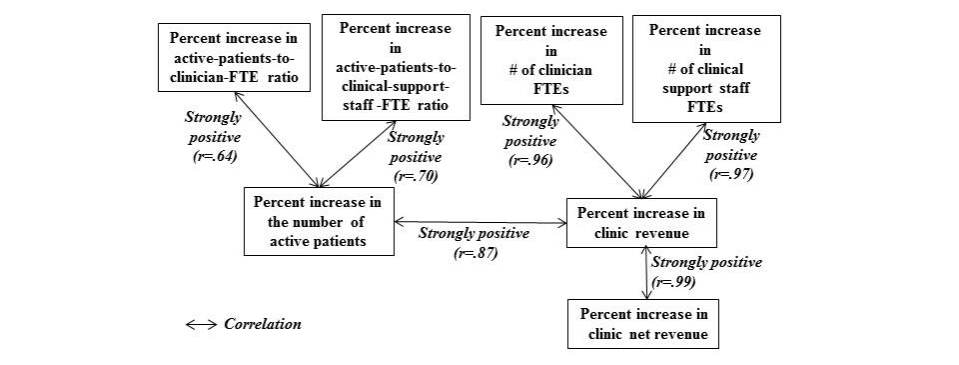
Correlations (r-values): clinic net revenue, clinic revenue, active patient count, clinician FTE count, clinical support staff FTE count, active-patients-to-clinician-FTE ratio, and active-patients-to-clinical-support-staff-FTE ratio.

Percent increase in the number of active patients showed strong positive correlations with percent increases in active-patients-to-clinician-FTE ratio (*r*=.64) and in active-patients-to-clinical-support-staff-FTE ratio (*r*=.70), as shown in Figure 4.

These correlations, together with the nonlinear percent changes summarized in Figure 3, suggest that the increased efficiency in the post-EHR period contributed to a clinic’s ability to accommodate the increased number of active patients.

#### Percent Changes in Number of Active Patients and Revenue After Implementation—Not Linearly Proportional to One Another

The percent increase in clinic revenue was also not linearly proportional to the percent increase in the number of active patients—an average increase of 76% versus an average increase of 56%, respectively. In addition to the increase in the number of active patients, there seem to be other factors that contributed to clinic revenue increase in the post-EHR period (detailed analysis on the impact of EHR on the sampled clinics’ billing patterns and revenue management processes, required to identify the contributing elements of the greater than linear increase in clinic revenue over the increase in patient count, is beyond the scope of the study).

The study also finds that percent increase in clinic net revenue was not linearly proportional to percent increase in clinic revenue. The average additional 13% increase in clinic net revenue (89%, which is 13% above the clinic average revenue of 76%) is attributable to the enhanced operational efficiency in the post-EHR period, which suggests the relative cost-savings effect after the EHR implementation.

#### Sign Test Results

We further tested the financial and operational impact of EHR in the post-EHR period, in order to assess the degree to which these findings could be extended to the population of clinics implementing EHR. The sign test, as opposed to *t* test, was adopted because the sample size was less than 30 and because the distributions shown in the data were not normal, with a high degree of skewness in most cases. The sign test results shown in Table 3 suggest, with a 95% confidence level, that the median percent change in clinic net revenue between the pre-EHR and post-EHR periods is positive in the population of the primary care clinics implementing EHR (sign test M=7.5 with *P*<.001).

**Table 3:** Summary of statistical analysis of change in a clinic’s operational efficiency and financial performance between between the pre-EHR and post-EHR periods

Percent changes between the pre-EHR and post-EHR periods	Average		SD		Median		M		Sign test, *P* value	
Percent change in clinic net revenue	89%		203%		23%		7.5		<.001	
Percent change in the number of active patients	56%		119%		10%		5.0		.006	
Percent change in active-patients-to-clinician-FTE ratio	27%		53%		9%		6.5		<.001	
Percent change in the number of clinician FTEs	22%		82%		0%		0.0		1.00	
Percent change in the number of clinical support staff FTEs	37%		75%		0%		3.0		.07	
Percent change in active-patients-to-clinical-support-staff-FTE ratio	10%		29%		4%		2.5		.277	

The same conclusions can be made for the median percent changes in the active-patients-to-clinician-FTE ratio and in the number of active patients in the same periods (M=6.5 and M=5, respectively). However, for the median percent changes with respect to the number of clinician FTEs, the number of clinical support staff FTEs, and the active-patients-to-clinical-support-staff-FTE ratio, we could not reject with a 95% confidence level the null hypothesis of no change after EHR implementation.

The correlation coefficients shown in Figure 4 and sign test results summarized in Table 4 suggest that the increase in the active patient count may not be the only factor that contributed to an average break-even point of 10 months upon EHR implementation. Percent increases in the number of active patients, in the active-patients-to-physician-FTE ratio, and in clinic net revenue appear to be positively associated with the EHR implementation, likely contributing substantially to an average break-even point of 10 months.

#### Analysis of Variance in Realizing Financial Performance—Key Factors

Study participants reported improvements in their ability to manage patient information after the implementation of EHR systems, citing improved ability to manage results such as obtaining test results from laboratories and following the results of an investigation over time (64%, 11/17 clinics). Respondents also reported an improved ability to seek out specific information from patient records (57%, 10/17 clinics), and access complete, up-to-date patient charts and review patient problems (43%, 6/15 clinics). See Table 4 for key EHR impacts expressed by study participants.

**Table 4:** Impact of EHR on clinic practices identified by study participants.

Categories	Participant comments
A. Impact of EHR on a clinic’s ability to manage results	“We receive results electronically and can graph them; graphs help ‘engage’ the patient.”
	“Direct to physician lab results has very positive effect on physician efficiency and patient care.”
B. Impact of EHR on a clinic’s ability to seek out specific information from patient records	“Complete chart is always available, anywhere which affects patient safety and means better care.”
	“Integration of information for referral requests is a great benefit.”
	“Billing codes are up-to-date. (And) billing is automatic by the doctor inside encounter note, which simplifies billing and is easier to manage reconciliation. No missed billing opportunities.”
C. Impact of EHR on a clinic’s ability to prepare patient encounter	“Review of patient information prior to encounter is greatly facilitated.”
	“Easier to prepare for encounter; maintenance of problem list /summary is much easier”
	“Immediate access to patient information—no lost files.”

Some primary care clinics did better than others in using EHR and achieving faster break-even from EHR investment, which can be observed in Tables 1 and 3. To gain insights into key differences between those clinics that were highly successful and those less successful in realizing a positive ROI from EHR, we conducted regression analysis on break-even point as the outcome variable. We used the codes identified through the coding process of the qualitative data as a part of the explanatory variables to estimate their relationships with the outcome variable “break-even point”. As summarized in Table 5, the regression analysis suggests four statistically significant factors impacting the return on EHR investment, that is, the time required to achieve cost recovery from an investment in EHR.

**Table 5:** Significant linear regression results of the outcome “break-even point” with explanatory variables (break-even point was log-transformed to approach a Normal distribution).

Explanatory variable	Variable values	Regression coefficient	Standard error of coefficient	*P* value	*r* ^2^
(a) Age of EHR: Months between Jan 1, 2013, and EHR implementation start date	Number of months	0.03	0.01	.049	.64
(b) e-Prescriptions complying with national standards	0 (No) to 1 (Yes)	-1.32	0.34	.006	.50
(c) Extent to which EHR complies to national standards	Continuous (from 0 to 10)	-0.19	0.07	.038	.54
(d) Process change: Use of flow sheets	0 (No) to 1 (Yes)	-1.29	0.46	.022	.68

Note that in Table 5, the regression coefficient of an explanatory variable with a negative value indicates faster recovery of the EHR investment (ie, a shorter time required to achieve cost recovery from an EHR investment), while a positive value implies slower recovery of the EHR investment (ie, a longer time required to achieve cost recovery from an EHR investment).

#### Age of Electronic Health Record Systems

The first result to note in item (a) of Table 5 is that older EHR implementations, in particular those implemented in 2004-2005, were slower to recover their investment, even though they still achieved a break-even point. This result suggests that the newer the EHR, the sooner a positive ROI can be achieved. The earlier EHR systems used by these clinics were less user-friendly and required longer training cycles for the users, which may explain why clinics with these earlier systems took longer to recoup their financial investment.

#### Compliance With National Standards

The second and third results, shown in items (b) and (c) of Table 5, suggest a positive link between the ROI indicator and the compliance with national standards such as codes representing prescription drugs. There was an improvement in clinics’ compliance with national standards and ability to comply with evidence-based medicine. This improvement was related to the age of the EHR system used by the clinics. Newer EHR implementations may be more likely to comply with national standards, given that the newer EHRs are likely to support national standards better.

#### Use of Flow Sheets and Ability to Manage Patient Information

Finally, clinics using EHR flow sheets scored consistently better times to break even, shown in item (d) of Table 5. Clinics reported the use of flow sheets, or structured data collection forms, as a mechanism for compliance to evidence-based medicine. The use of flow sheets in EHRs provides advanced features such as those related to the automatic maintenance of patient problem lists and pharmacological profiles. These enhanced features contribute directly to the physician’s efficiency by eliminating the time that would otherwise be spent manually maintaining these lists—a task that can be time-consuming, highly repetitive, and labor-intensive to maintain with consistency in a paper-based environment. The availability of up-to-date lists makes patient encounter preparation easier and more rapid, as the necessary information is available at a glance.

### Analysis of Electronic Health Record Functionalities Used in Primary Care Clinics

Our study finds that despite the limited use of EHR functionalities and limited interoperability, the sampled clinics achieved overall positive operational and financial performance. Table 6 summarizes the data we gathered on EHR functionalities, frequency of use, and ease of use.

Most frequently and routinely used EHR functionalities were related to medication management. Health information exchange and patient engagement portal functionalities saw no significant use (the investigation of why these functionalities were not used is beyond the scope of this study).

Respondents stressed that it typically takes a few months to understand any particular EHR function sufficiently to effectively introduce it in their clinical practices. This finding, coupled with the finding that despite the limited use of EHR functionalities the clinics achieved overall positive improvement in operational and financial performance in the post-EHR period, suggests that a clinic’s ability to embed particular EHR functionalities in their workflow and make use of these functionalities in their day-to-day clinical practices is of more importance in realizing a positive ROI from EHR implementation than implementing an EHR software package with the maximum number of features and functionalities.

**Table 6:** EHR functionalities and utilization reported during the study period.

EHR functionalities		% of clinics answering in the affirmative
**User Interface: Does the EHR system currently in use at this clinic have any of the following user interface technologies? (N=17)**		
	Alternative presentation formats for clinical information	100.0
	Support for guideline-based data collection and treatment	94.1
	Support for multiple platform access	88.2
	Support for context sensitive alerts, warnings, and guidance	70.6
	Clinical notes capture in narrative form	23.5
**Listing functionality: With the EHR system you currently have, how easy is it for you (or staff in your practice) to generate the following information about your patients? (N=17)**		
	List of all medications taken by an individual patient	100.0
	Provide patients with clinical summaries for each visit	88.2
	List of all laboratory results for an individual patient	88.2
	List of patients by diagnosis (eg, diabetes or cancer)	82.4
	List of patients who are due or overdue for tests or preventive care	76.5
	List of all patients taking a particular medication	76.5
	List of patients by laboratory result (eg, HbA1C>9.0)	52.3
**Reminder functionality: Are the tasks routinely performed for patients at your site using EHR? (N=17)**		
	Clinicians receive a reminder for guideline-based interventions and/or screening tests	58.8
	Clinicians receive an alert or prompt to provide patients with test results	41.2
	Patients are sent reminder notices when it is time for regular preventive or follow-up care	35.3
	All laboratory tests ordered are tracked until results reach clinicians	29.4
**Does your site and the clinicians that practice in your site use the EHR system to facilitate any of the following workflow activities (N=16)**		
	Electronic prescribing of medication	93.3
	Electronic prompts about a potential problem with drug dose or drug interaction	87.5
	Electronic receipt of laboratory results integrated into the EHR system (not scanned)	62.5
	Electronic ordering of laboratory tests	43.8
	Electronic referring to specialists	37.5
	electronic transferring of prescriptions to a pharmacy	6.7
**Health information exchange functionalities: Can you electronically exchange the following with any doctors outside your practice? (N=16)**		
	Electronic exchange outside practice: patient clinical summaries	25.0
	Electronic exchange outside practice: laboratory and diagnostic tests	18.8
**Patient engagement functionality: Please indicate whether the EHR system in use at your site allows patients to… (N=17)**		
	Access alcohol consumption advice online	11.8
	Access advice for informal caregivers online	11.8
	Email about a medical question or concern	11.8
	Access dietary advice online	11.8
	Access advice on physical activity online	11.8
	Access advice on self-management of chronic conditions online	11.8
	Access smoking cessation advice online	11.8
	Request appointments online	5.9
	View a list of medications (current and past) online	5.9
	View other components of their chart (current and past) online	0.0
	View medical imaging results (current and past) online	0.0
	Request refills for prescriptions online	0.0
	View test results (current and past) online	0.0
**Interoperability: Were any of the following INTEROPERABILITY technologies implemented in the EHR system currently in use at this site? (N=17)**		
	Diagnoses are coded using international standards	94.1
	Medications and pharmacological profiles are coded to national standards	82.4
	Patient records are supported by standards-based data migration technology	50.0
	ePrescriptions comply with national standards	52.9
	Patient Identifier is based on national or jurisdictional standard	58.8
	Patient Identifier is supported by aliasing technology to achieve positive ID across systems	37.5
	Findings are coded using international standards	58.8
	Communications with other clinics and institutions use international standards	31.3
	Investigations, referrals, and imaging requests make use of order tracking technology	35.3

## Discussion

### Principal Findings

This study aimed to complement current insights into the cost recovery concerns related to EHR investments by considering the research question “When implemented in primary care practices, what will be the ROI from EHR?”. The study finds that primary care clinics can realize a positive ROI from the implementation of EHR. Our analysis offers evidence that the increases in net revenue, in the active-patients-to-clinician-FTE ratio, and in the number of active patients are positively associated with the EHR implementation, likely contributing substantially to an average break-even point of 10 months.

In addition, the analysis conducted to understand the variances in financial and operational performance among the sampled clinics provides insights into key differences between those clinics that were highly successful and those less successful in realizing a positive ROI from EHR. Some clinics seem to be more innovative than others in using EHR in their practices to achieve significantly better operational and financial results. The analysis suggests that a clinic’s ability to take advantage of EHR to support process changes has a significant effect on the time required to achieve cost recovery from an investment in EHR. In particular, the clinics that were successful in realizing faster time to break even were better at using EHR in workflow areas involving patient information—such as maintaining patient problem lists, managing test results, and complying with national coding standards, all of which make patient encounter preparation easier and more rapid. We also find that the clinics achieved positive financial performance, even though not all EHR functionalities were used. The alignment of EHR functionalities with clinic workflow plays an important role in achieving positive operational and financial results with EHR. Identified as particularly important EHR-product improvements that would ease adoption of workflow changes are automations that assist clinicians, clinical support staff, and administrative staff both in the overall management of the practice and within the patient encounter, as well as consistent and comprehensive compliance with national standards such as national drug coding standards.

### Implications for Practitioners and Managers in Primary Care

The knowledge gained from this ROI study on EHR is important to practicing primary care physicians who are concerned about how they will fare financially upon investing in EHR, as they face ever increasing pressure to transition from their paper-based records to electronic systems. This study provides evidence to practitioners in primary care that investment in EHR can be a sound decision with a reasonable cost recovery time frame, while providing immediate opportunities for increased operational efficiency and the potential for further improvements in clinic performance and benefits realization from EHR. Practitioners in primary care who are considering the investment in EHR should note the important relationship between EHR functionality, clinic workflow change, and a positive ROI from EHR implementation. Positive ROI does not happen automatically upon implementing an EHR package, and a clinic’s ability to leverage EHR for process changes plays a role in achieving a positive ROI.

### Implications for Policy Makers

This study’s finding on increased active patient count and clinic operational efficiency after the EHR implementation, in particular with respect to improvement in the active-patients-to-clinician-FTE ratio, offers the possibility that EHR can play a role in addressing the shortage in family physicians. As primary care clinics implement EHR systems and discover better ways to take advantage of EHR in their practices, a key question will be how to incorporate such learnings and deliver enhanced EHR products back into the clinics to realize the full potential of EHR. Policies that enable the establishment of a closed-loop feedback mechanism between EHR vendors and health care providers could facilitate targeted enhancements to EHR systems. In addition, policies that provide support to help primary care practices successfully make EHR-enabled changes, such as support of workflow optimization with an EHR system that would ease adoption, could not only facilitate the realization of positive ROI but also help address the shortage in family physicians.

### Future Research

**Figure 5:**
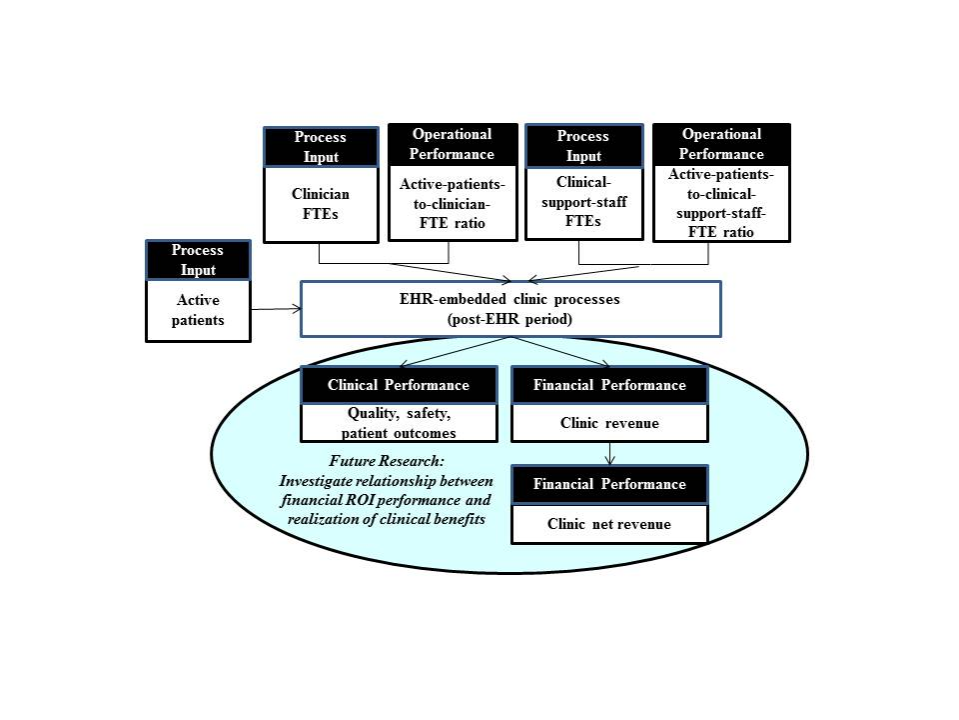
Future research: investigate the relationship between return on EHR investment and clinical benefits realization from EHR implementation.

Some of the factors identified in this research as key factors impacting the realization of a positive ROI from EHR implementation, such as improved access to up-to-date patient charts and improved ability to obtain test results from laboratories and follow the results of an investigation over time, have implications to quality of care and patient safety. Thus, future research will be to investigate the relationship between financial ROI and realization of clinical benefits of EHR such as quality, safety, and patient outcomes, as depicted in Figure 5. Other research should include a study to identify best practices for implementing and using EHR, with concrete examples of success factors and failure factors as well as ways to tailor these best practices relevant to particular clinic situations. In addition, panel analysis, which deals with two-dimensional panel data (cross sectional and times series) [35], can be conducted with the cohort of primary care clinics to understand the effect of learning curve on a clinic’s ability to realize positive ROI and non-financial, clinical benefits from EHR implementation. Knowledge gained from such studies could facilitate EHR adoption and subsequent benefits realization in primary care practices.

### Limitations

The principal limitation of this study is that the number of primary care clinics examined was limited, due mainly to time constraints of clinics to participate in the study and scarcity of suitably detailed operational and financial data necessary for ROI calculation. For the clinics recruited to the study, the most limiting factor was that of collecting a complete picture of the cost and benefits needed to assess an ROI from EHR implementation. This was due mainly to the absence of standardized financial and business-case approaches to the governance of these independent organizations. The insights gained from the participants in our study, however, provide salient insights into the impact of EHR investment to facilitate the EHR adoption across practicing primary care physicians, with information on time required to achieve cost recovery from an EHR investment and on principal factors impacting cost-recovery performance.

## Abbreviations

EHR: electronic health records
FTE: full time equivalent
IT: information technology
ROI: return on investment
